# Assessing responsiveness of generic and specific health related quality of life measures in heart failure

**DOI:** 10.1186/1477-7525-4-89

**Published:** 2006-11-24

**Authors:** Dean T Eurich, Jeffrey A Johnson, Kimberly J Reid, John A Spertus

**Affiliations:** 1 Institute of Health Economics, Edmonton, Alberta, Canada; 2 Department of Public Health Sciences, University of Alberta, Edmonton, Alberta, Canada; 3 Saint Lukes Health Centre, Kansas City, Missouri, US; 4 Mid-America Heart Institute and University of Missouri, Kansas City, Missouri, US

## Abstract

**Background:**

Responsiveness, or sensitivity to clinical change, is an important consideration in selection of a health-related quality of life (HRQL) measure for trials or clinical applications. Many approaches can be used to assess responsiveness, which may affect the interpretation of study results. We compared the relative responsiveness of generic and heart failure specific HRQL instruments, as measured both by common psychometric indices and by external clinical criteria.

**Methods:**

We analyzed data collected at baseline and 6-weeks in 298 subjects with heart failure on the following HRQL measures: EQ-5D (US, UK, and VAS Scoring), Kansas City Cardiomyopathy Questionnaire (KCCQ) (Clinical and Overall Summary Score), and RAND12 (Physical and Mental Component Summaries). Three external indicators of clinical change were used to classify subjects as improved, deteriorated, or unchanged: 6-minute walk test, New York Heart Association (NYHA) class, and physician global rating of change. Four responsiveness statistics (T-test, effect size, Guyatt's responsiveness statistic, and standardized response mean) were used to evaluate the responsiveness of the select measures. The median rank of each HRQL measure across responsiveness indices and clinical criteria was then determined.

**Results:**

Average age of subjects was 60 years, 75 percent were male, and had moderate to severe heart failure symptoms. Overall, the KCCQ Summary Scores had the highest relative ranking, irrespective of the responsiveness index or external criterion used. Importantly, we observed that the relative ranking of responsiveness of the generic measures (i.e. EQ-5D, RAND12) was influenced by both the responsive indices and external criterion used.

**Conclusion:**

The disease specific KCCQ was the most responsive HRQL measure assessing change over a 6-week period, although generic measures provide information for which the KCCQ is not suitable. The responsiveness of generic HRQL measures may be affected by the index used, as well as the external criterion to classify patients who have clinically change or remained stable.

## Background

In chronic medical conditions, small changes in clinical status must be readily identifiable to monitor patients' progress and to modify treatment strategies, if necessary [1]. In heart failure, for example, numerous clinical indicators are employed to monitor patients' health status over time, including physician assessments (e.g., New York Heart Association (NYHA) classification system), exercise capacity (e.g., six-minute walk test), fluctuations in body weight, and biomarkers [2]. Often, however, changes in patients' own perceptions of their health status may not be readily apparent to the clinician or may not be manifested in a manner that easily lends itself to these assessments. As a result, self-reported health-related quality of life (HRQL) measures are increasingly being used to provide complementary and additional insight into the health status of a patient or patients [3-6].

HRQL measures have been commonly used in the clinical trial setting in the evaluation of new treatment strategies, and more recently are even used as the primary outcome assessment for clinical trials [7,8]. When used as the primary outcome, identification and quantification of subtle changes in a patients' health status is critical since the success or failure of the trial depends entirely on the HRQL measure. It is therefore essential that the HRQL measure be sensitive to small, but important, changes to determine if the treatment under study is effective or potentially harmful to the patient.

Several disease and generic measures of HRQL have been used in both clinical practice and in clinical trials of patients with heart failure. To accurately capture these changes in health status over time, the HRQL measure must have evidence of longitudinal validity or 'responsiveness' [9]. Responsiveness refers to the ability of a HRQL measure to capture *true *underlying change in the patients' health status over time [9]. Two approaches are commonly used to assess the responsiveness of HRQL measures. The first method, the distributional approach, imparts meaning to the HRQL score by evaluating the changes in the HRQL scores and their associated variability (i.e., standard deviation). Often distributional based methods establish the responsiveness of a HRQL measure by the degree of 'statistical significance' associated with the change score. Importantly, however, the interpretability of the data is completely dependent on the variability of the data and a 'statistically significant' change may not necessarily constitute a clinically important change (or vice-versa), thereby limiting their ability to evaluate responsiveness. The second method, anchored-based approaches, compares the changes in HRQL scores to other clinically meaningfully markers or anchors. Anchor-based approaches are often easier to interpret for clinical audiences than the distributional-based approaches. Importantly, however, the external anchor chosen must itself be a valid measure of clinical change.

Several factors may therefore influence the responsiveness of a HRQL measure, including, but not limited to, the content of the measure (i.e., disease-specific versus generic), the validity of the measure, the error associated with the HRQL scores, the indices used to determine the responsiveness (e.g., T-statistics, effect sizes, etc) and the external criterion or 'gold standard' used to identify subjects as changed or not changed. When considering evidence of responsiveness, it is important to consider the extent to which these factors affect reported estimates of responsiveness [9].

To provide empirical evidence to support this issue, and to assist in the selection and interpretation of health status measures for heart failure, we analyzed longitudinal HRQL data in patients with heart failure to evaluate the relative responsiveness of selected disease-specific and generic HRQL measures. We explicitly compared their relative performance as measured by common responsiveness indices and by different external clinical criteria for change.

## Methods

### Sample

Patients with heart failure were recruited through the Cardiovascular Outcomes Research Consortium across 14 medical center outpatient departments in the United States and Canada [2]. All subjects were 30 years of age or older with documented left ventricular systolic dysfunction (left ventricular ejection fraction < 0.40). There were no exclusions, particularly with respect to the upper age limit. The subjects included in the study were typical of heart failure subjects in an outpatient setting. This was a cohort study aimed at evaluating the random changes observed in heart failure patients in the outpatient setting. No specific intervention was studied in these patients during the follow-up period.

### HRQL and clinical measures

Patients completed several HRQL questionnaires at baseline, including the RAND12, EQ-5D, and the Kansas City Cardiomyopathy Questionnaire (KCCQ).

The RAND12 is a short, well-validated, generic measure of health status [10-12]. The patients overall physical and mental health status was evaluated using the United States (US) population standardized Physical Component Summary (PCS) and the Mental Component Summary (MCS) scores [10,11].

The EQ-5D is a 5-item self-administered utility measure [13]. In addition to the 5 health state items, the EQ-5D also contains a visual analog scale (EQ-VAS). Utility scores are generated using the time-trade off approach where the responses to the five items are valued based on general population valuation scores. Health state valuations are available for both the United Kingdom (UK) [13] and more recently the US [14,15].

The KCCQ is a 23-item heart failure specific questionnaire. The KCCQ has domains on physical limitations, heart failure specific symptoms (e.g., swelling, shortness of breath, fatigue), quality of life, social impact of the disease, and patients' assessments of their disease knowledge or self-efficacy [16]. The psychometric properties of the measure have been previously established [2,16]. In addition to domain scores, the KCCQ generates two summary measures, the KCCQ Clinical Summary Score (capturing patients' physical function and symptoms) and KCCQ Overall Summary Score (including the physical and social function, symptoms and quality of life domains). These summary scores were used for all analyses involving the KCCQ.

In addition to the HRQL questionnaires, several clinical assessments were also completed by the residing cardiologist at baseline. Specifically, the patients baseline New York Heart Association (NYHA) classification was assessed and patients completed a six-minute walk (6 MW) test according to a standardized protocol [17]. All patients returned to the medical center after approximately 6 weeks for repeat assessment by the cardiologist. All measures, including HRQL questionnaires (i.e., RAND12, EQ-5D, and KCCQ), NYHA classification, and 6 MW tests were re-evaluated.

### Criteria for clinical change

There is no universally accepted gold standard for identification of clinical change in patients with heart failure. As a result, change in the patients' clinical status was assessed using several criteria. First, cardiologist's assessment of the patients' NYHA classification at baseline and at the 6-week follow-up was used. Subjects were classified as improving two NYHA classes (e.g., improving from NYHA class IV to NYHA class II), improving one NYHA class, no change in NYHA class, and deteriorating one NYHA class from the baseline to 6-week follow-up. No subjects deteriorated by two NYHA classes during the 6-week period.

Second, the resident cardiologist, blinded to the subjects self-reported HRQL and the 6 MW test results, completed a previously validated global rating of change assessment from the baseline to the 6-week follow-up visit [2,18]. This provides a 15-point Likert scale ranging from extremely worse (-7), no change (zero), to extremely better (+7). Subjects were classified into 5 mutually exclusive change categories: substantially improved (+7, +6, +5), moderately improved (+4,+3,+2), no change (+1, 0, -1), moderately deteriorated (-2, -3, -4), and substantially deteriorated (-5, -6, -7).

Finally, the difference from baseline to 6-weeks in distanced traveled in the 6 MW test was recorded. This difference was categorized into 7 mutually exclusive categories of clinical change according to previous research [2]: substantially improved (≥ +100 meters); moderately improved (+50 to +99 meters), small improvement (+25 to +49 meters), no change (+24 to -24 meters), small deterioration (-25 to -99 meters), moderately deteriorated (-100 to -199 meters), and substantial deterioration (≤ -200 meters).

### Analysis

Mean change scores in the HRQL measures were calculated by subtracting the baseline score from the 6-week follow-up data. Responsiveness indices, including T-statistic (mean change divided by standard deviation for total group), effect size (ES) (mean change divided by the standard deviation of the baseline score), the Guyatt's responsiveness statistic (GRS) (mean change divided by the standard deviation of change in subjects who remained unchanged), and the standardized response mean (SRM) (mean change divided by the standard deviation of the change score) [9], were calculated for each of the HRQL measures (i.e., RAND12–MCS and PCS Scores, EQ-5D–US, EQ-5D–UK, and EQ–VAS, and the KCCQ–Overall Summary Score and Clinical Summary Score) between patients who changed and remained stable from baseline to 6-weeks [9]. The responsiveness indices were calculated for each of the HRQL measures according to the degree of change as identified by the three primary external indicators of heart failure status change.

To facilitate comparison, the median rank of each HRQL measure was determined across each of the four responsiveness indices [19]. In order for a HRQL measure to be a valid measure of clinical change, the measure must be capable of capturing both improvements and deterioration in clinical status. As a result, categories for improvement and deterioration were combined to provide an overall single median rank, depicting the overall relative responsiveness of the HRQL measure to changes in heart failure clinical status. Of note, the HRQL scores in subjects who remained stable (i.e., no change categories) were not included in the calculation of the overall single median rank of responsiveness.

## Results

A total of 476 subjects were enrolled in the study and provided baseline and 6-week follow-up [2]. Of these subjects, 298 had complete data and were included. Subjects included in the analysis did not differ in age, sex, body mass index, comorbidities, heart failure symptoms, or baseline health status compared to the total cohort (p > 0.05 for all comparisons). Subjects were mainly elderly, male, overweight, and had a significant history of other comorbidities (Table 1). Most subjects were in NYHA class II and III heart failure indicating moderate to severe heart failure symptoms. Overall, baseline scores indicate subjects reported substantial deficits in their HRQL consistent with the clinical indicators of their condition (Table 2).

**Table 1:** Baseline Characteristics

**Characteristic (n = 298)**	**Number (%) or Mean ± SD**
**Demographics**	
Age	60 ± 13
Sex (male)	223 (75)
Race	
Caucasian	216 (73)
African-American	69 (23)
Other	13 (4)
Body mass index	29.0 ± 6.5*
**Co-Morbidities**	
History Hypertension	159 (53)
History of angina	66 (22)
History of myocardial infarction	112 (38)
History of percutaneous coronary intervention	41 (14)
History of coronary artery bypass graph	82 (28)
History of hyperlipidemia	135 (45)
History of diabetes	99 (33)
History of renal failure	15 (5)
History of COPD/Asthma	56 (19)
**Heart Failure**	
Etiology †	
- Ischemia	146 (50)
- Hypertensive	25 (8)
- Valvular heart disease	8 (3)
- Other	110 (38)
Ejection fraction	25 ± 8.1
Heart Rate	73 ± 14
Systolic blood pressure	121 ± 24
Diastolic blood pressure	69 ± 13
Receiving ACE inhibitors	242 (81)
Receiving beta-blockers	224 (75)
Receiving spironolactone	106 (36)

*–1 subject missing data; †–9 subjects missing data; COPD -Chronic Obstructive Pulmonary Disease

**Table 2:** Baseline Health Status and Clinical Measures

**Health Status Measure (n = 298)**	**Number (%) or Mean ± SD**
RAND12–PCS	35.0 ± 10.7
RAND12–MCS	48.4 ± 11.4
EQ5D–UK Valuation	0.66 ± 0.26
EQ5D–US Valuation	0.74 ± 0.17
EQ–VAS	62.6 ± 20.5*
KCCQ–Clinical Summary Score	65.8 ± 23.3
KCCQ–Overall Summary Score	61.4 ± 24.1
NYHA Class	
I	34 (11)
II	129 (43)
III	122 (41)
IV	13 (4)
Six minute walk distance (m)	305 ± 112

*–7 people missing baseline values

### Classification according to clinical criteria

Upon follow-up (average 6 ± 2 weeks), 52 (17%) subjects were classified as improved according to the NYHA class (Table 3), 60 (20%) subjects improved according to the global rating of change (Table 4), and 101 (34%) subjects improved according to the 6 MW test (Table 5). Conversely, 40 (13%) subjects were classified as deteriorated according to the NYHA class, 32 (11%) subjects deteriorated according to the global rating of change, and 83 (28%) subjects deteriorated according to the 6 MW test. Overall, 206 (69%) subjects were classified as not changed according to both the NYHA class (Table 3) and the global rating of change (Table 4) and 114 (38%) according to 6 MW test (Table 5).

**Table 3:** Baseline, 6 week, and mean change scores according to change in health status according to the external criterion: New York Heart Association Classification

	**n**	**Baseline Score ± Standard Deviation**	**6 Week Score ± Standard Deviation**	**Mean Change Score ± Standard Deviation**
		**EQ5D–US Scoring**		
*+2 NYHA Classes*	2	0.78 ± 0.11	0.82 ± 0.06	0.04 ± 0.05
*+1 NYHA Class*	50	0.76 ± 0.16	0.77 ± 0.16	0.01 ± 0.13
*No change in NYHA Class*	206	0.74 ± 0.18	0.77 ± 0.16	0.03 ± 0.13
*-1 NYHA Class*	40	0.73 ± 0.18	0.74 ± 0.17	0.01 ± 0.12
*-2 NYHA Classes*	0	-	-	-
		**EQ5D–UK Scoring**		
*+2 NYHA Classes*	2	0.75 ± 0.19	0.79 ± 0.14	0.04 ± 0.05
*+1 NYHA Class*	50	0.68 ± 0.25	0.70 ± 0.24	0.02 ± 0.19
*No change in NYHA Class*	206	0.66 ± 0.27	0.71 ± 0.22	0.05 ± 0.20
*-1 NYHA Class*	40	0.65 ± 0.27	0.65 ± 0.25	0.00 ± 0.19
*-2 NYHA Classes*	0	-	-	-
		**EQ–VAS**		
*+2 NYHA Classes*	2	71.00 ± 12.73	77.50 ± 10.61	6.50 ± 2.12
*+1 NYHA Class*	46	59.83 ± 21.05	62.10 ± 21.32	1.09 ± 23.84
*No change in NYHA Class*	198	63.95 ± 20.05	65.74 ± 20.62	1.45 ± 16.17
*-1 NYHA Class*	37	58.88 ± 21.88	60.38 ± 22.31	2.68 ± 18.56
*-2 NYHA Classes*	0	-	-	-
		**KCCQ OVERALL SUMMARY SCORE**		
*+2 NYHA Classes*	2	72.27 ± 1.66	78.65 ± 12.52	6.38 ± 14.18
*+1 NYHA Class*	50	60.87 ± 24.30	65.65 ± 21.39	4.77 ± 15.60
*No change in NYHA Class*	206	61.87 ± 24.25	63.84 ± 24.23	1.97 ± 11.49
*-1 NYHA Class*	40	58.93 ± 23.63	54.56 ± 22.48	-4.37 ± 13.05
*-2 NYHA Classes*	0	-	-	-
		**KCCQ CLINCAL SUMMARY SCORE**		
*+2 NYHA Classes*	2	73.18 ± 8.47	76.04 ± 10.31	2.86 ± 18.78
*+1 NYHA Class*	50	65.98 ± 23.24	71.10 ± 18.64	5.11 ± 15.75
*No change in NYHA Class*	206	65.85 ± 23.63	67.50 ± 23.74	1.65 ± 11.59
*-1 NYHA Class*	40	64.70 ± 22.37	59.59 ± 22.02	-5.1 ± 11.51
*-2 NYHA Classes*	0	-	-	-
		**RAND12 PCS**		
*+2 NYHA Classes*	2	40.15 ± 9.86	37.39 ± 6.53	-2.77 ± 3.33
*+1 NYHA Class*	50	33.69 ± 10.4	35.08 ± 9.87	1.40 ± 9.20
*No change in NYHA Class*	206	35.69 ± 11.09	36.58 ± 11.02	0.89 ± 6.47
*-1 NYHA Class*	40	32.71 ± 8.89	33.02 ± 8.28	0.31 ± 6.22
*-2 NYHA Classes*	0	-	-	-
		**RAND12 MCS**		
*+2 NYHA Classes*	2	45.44 ± 22.36	51.01 ± 17.25	5.57 ± 5.11
*+1 NYHA Class*	50	50.40 ± 9.98	49.43 ± 10.61	-0.97 ± 10.19
*No change in NYHA Class*	206	48.03 ± 11.55	48.45 ± 11.24	0.41 ± 8.35
*-1 NYHA Class*	40	48.22 ± 12.18	46.00 ± 11.80	-2.21 ± 9.89
*-2 NYHA Classes*	0	-	-	-

**Table 4:** Baseline, 6 week, and mean change scores according to change in health status according to the external criterion: Global Rating of Change

	**n**	**Baseline Score ± Standard Deviation**	**6 Week Score ± Standard Deviation**	**Mean Change Score ± Standard Deviation**
		**EQ5D–US Scoring**		
*Substantial Increase (+ 5,6,7)*	7	0.74 ± 0.18	0.88 ± 0.12	0.14 ± 0.17
*Moderate Increase (+2,+3,+4)*	53	0.76 ± 0.15	0.79 ± 0.13	0.02 ± 0.12
*No Change (-1,0,+1)*	206	0.74 ± 0.18	0.77 ± 0.16	0.03 ± 0.13
*Moderate Decrease (-2,-3,-4)*	30	0.74 ± 0.15	0.73 ± 0.12	-0.01 ± 0.11
*Substantial Decrease (-5,-6,-7)*	2	0.46 ± 0.19	0.43 ± 0.24	-0.03 ± 0.05
		**EQ5D–UK Scoring**		
*Substantial Increase (+ 5,6,7)*	7	0.65 ± 0.28	0.86 ± 0.16	0.21 ± 0.26
*Moderate Increase (+2,+3,+4)*	53	0.70 ± 0.23	0.73 ± 0.18	0.03 ± 0.20
*No Change (-1,0,+1)*	206	0.66 ± 0.27	0.70 ± 0.24	0.04 ± 0.19
*Moderate Decrease (-2,-3,-4)*	30	0.66 ± 0.22	0.65 ± 0.18	-0.01 ± 0.19
*Substantial Decrease (-5,-6,-7)*	2	0.27 ± 0.34	0.22 ± 0.42	-0.05 ± 0.07
		**ED–VAS**		
*Substantial Increase (+ 5,6,7)*	5	54.80 ± 34.28	75.71 ± 25.24	19.2 ± 22.26
*Moderate Increase (+2,+3,+4)*	51	64.66 ± 20.96	67.25 ± 20.37	2.90 ± 23.39
*No Change (-1,0,+1)*	196	63.05 ± 20.27	65.16 ± 20.52	1.70 ± 15.92
*Moderate Decrease (-2,-3,-4)*	29	58.07 ± 18.91	55.00 ± 20.31	-2.66 ± 15.64
*Substantial Decrease (-5,-6,-7)*	2	55.00 ± 7.07	30.00 ± 14.14	-25.00 ± 21.21
		**KCCQ OVERALL SUMMARY SCORE**		
*Substantial Increase (+ 5,6,7)*	7	51.26 ± 30.57	70.98 ± 27.65	19.72 ± 17.24
*Moderate Increase (+2,+3,+4)*	53	63.79 ± 20.38	68.47 ± 18.43	4.69 ± 15.84
*No Change (-1,0,+1)*	206	62.33 ± 24.81	63.86 ± 24.16	1.52 ± 10.82
*Moderate Decrease (-2,-3,-4)*	30	55.54 ± 21.12	48.82 ± 19.80	-6.72 ± 11.61
*Substantial Decrease (-5,-6,-7)*	2	21.88 ± 8.84	13.28 ± 6.26	-8.59 ± 2.58
		**KCCQ CLINCAL SUMMARY SCORE**		
*Substantial Increase (+ 5,6,7)*	7	56.55 ± 31.08	74.40 ± 21.88	17.86 ± 16.53
*Moderate Increase (+2,+3,+4)*	53	69.24 ± 20.42	72.54 ± 16.59	3.30 ± 16.58
*No Change (-1,0,+1)*	206	66.38 ± 23.78	67.74 ± 23.71	1.36 ± 10.83
*Moderate Decrease (-2,-3,-4)*	30	60.46 ± 19.73	54.94 ± 18.96	-5.52 ± 11.55
*Substantial Decrease (-5,-6,-7)*	2	22.92 ± 14.73	14.58 ± 7.37	-8.33 ± 7.37
		**RAND12 PCS**		
*Substantial Increase (+ 5,6,7)*	7	29.29 ± 12.53	41.00 ± 13.79	11.71 ± 10.41
*Moderate Increase (+2,+3,+4)*	53	35.66 ± 10.99	36.66 ± 9.64	1.00 ± 7.82
*No Change (-1,0,+1)*	206	35.51 ± 10.81	36.34 ± 10.69	0.83 ± 6.43
*Moderate Decrease (-2,-3,-4)*	30	32.66 ± 8.00	31.00 ± 8.06	-1.66 ± 5.72
*Substantial Decrease (-5,-6,-7)*	2	17.76 ± 1.36	19.21 ± 2.99	1.45 ± 4.36
		**RAND12 MCS**		
*Substantial Increase (+ 5,6,7)*	7	54.23 ± 13.01	49.04 ± 12.92	-5.19 ± 10.47
*Moderate Increase (+2,+3,+4)*	53	48.41 ± 11.40	51.61 ± 10.00	3.20 ± 10.61
*No Change (-1,0,+1)*	206	48.73 ± 11.49	48.26 ± 11.27	-0.47± 8.11
*Moderate Decrease (-2,-3,-4)*	30	45.40 ± 10.54	42.98 ± 11.17	-2.41 ± 9.46
*Substantial Decrease (-5,-6,-7)*	2	44.56 ± 10.15	42.43 ± 7.13	-2.14 ± 3.03

**Table 5:** Baseline, 6 week, and mean change scores according to change in health status according to the external criterion: Six-Minute Walk Test (meters)

	**n**	**Baseline Score ± Standard Deviation**	**6 Week Score ± Standard Deviation**	**Mean Change Score ± Standard Deviation**
		**EQ5D–US Scoring**		
*<= -200*	7	0.69 ± 0.11	0.68 ± 0.14	-0.02 ± 0.09
*-199 to -100*	16	0.66 ± 0.24	0.68 ± 0.20	0.02 ± 0.12
*-99 to -25*	60	0.72 ± 0.17	0.75 ± 0.16	0.04 ± 0.15
*-24 to +24*	114	0.77 ± 0.17	0.77 ± 0.15	0.00 ± 0.11
*25 to 49*	33	0.77 ± 0.16	0.80 ± 0.13	0.04 ± 0.13
*50 to 99*	40	0.72 ± 0.18	0.78 ± 0.16	0.05 ± 0.14
>= 100	28	0.76 ± 0.16	0.80 ± 0.16	0.04 ± 0.11
		**EQ5D–UK Scoring**		
*<= -200*	7	0.59 ± 0.18	0.59 ± 0.22	0.00 ± 0.12
*-199 to -100*	16	0.53 ± 0.35	0.56 ± 0.32	0.03 ± 0.20
*-99 to -25*	60	0.62 ± 0.26	0.68 ± 0.24	0.06 ± 0.23
*-24 to +24*	114	0.70 ± 0.25	0.71 ± 0.22	0.01 ± 0.17
*25 to 49*	33	0.70 ± 0.23	0.75 ± 0.19	0.05 ± 0.17
*50 to 99*	40	0.63 ± 0.28	0.71 ± 0.23	0.08 ± 0.23
>= 100	28	0.69 ± 0.24	0.74 ± 0.24	0.05 ± 0.16
		**EQ–VAS**		
*= -200*	7	60.43 ± 15.04	50.71 ± 21.30	-9.71 ± 20.95
*-199 to -100*	15	58.38 ± 27.40	49.00 ± 31.12	-8.60 ± 23.21
*-99 to -25*	56	60.53 ± 19.33	63.92 ± 21.71	2.32 ± 18.94
*-24 to +24*	109	64.94 ± 19.06	66.13 ± 20.19	1.24 ± 17.59
*25 to 49*	31	66.41 ± 18.46	68.84 ± 17.37	2.52 ± 13.28
*50 to 99*	38	60.89 ± 23.83	65.60 ± 17.66	3.82 ± 17.46
>= 100	27	58.41 ± 22.41	64.86 ± 20.93	5.89 ± 15.84
		**KCCQ OVERALL SUMMARY SCORE**		
*<= -200*	7	45.44 ± 22.81	45.44 ± 22.81	-7.75 ± 13.19
*-199 to -100*	16	53.42 ± 24.82	53.42 ± 24.82	-5.00 ± 6.20
*-99 to -25*	60	61.54 ± 24.20	61.54 ± 24.20	0.08 ± 13.99
*-24 to +24*	114	62.82 ± 23.30	62.82 ± 23.30	1.47 ± 10.94
*25 to 49*	33	67.41 ± 23.69	67.41 ± 23.69	0.17 ± 12.09
*50 to 99*	40	59.35 ± 26.26	59.35 ± 26.26	3.74 ± 16.69
>= 100	28	59.48 ± 21.64	59.48 ± 22.89	10.3 ± 14.08
		**KCCQ CLINCAL SUMMARY SCORE**		
*<= -200*	7	45.64 ± 26.24	37.28 ± 25.84	-8.36 ± 11.76
*-199 to -100*	16	58.93 ± 22.54	55.89 ± 25.92	-3.03 ± 9.85
*-99 to -25*	60	65.01 ± 23.73	65.54 ± 23.94	0.53 ± 13.30
*-24 to +24*	114	67.07 ± 22.07	67.72 ± 21.66	0.65 ± 11.68
*25 to 49*	33	72.41 ± 22.48	72.39 ± 22.91	-0.02 ± 10.96
*50 to 99*	40	64.45 ± 25.65	68.61 ± 20.59	4.16 ± 13.72
>= 100	28	65.10 ± 22.37	73.41 ± 19.31	8.31 ± 14.24
		**RAND12 PCS**		
*<= -200*	7	25.49 ± 9.07	25.74 ± 6.88	0.25 ± 8.15
*-199 to -100*	16	32.28 ± 12.07	34.7 ± 12.05	2.42 ± 5.58
*-99 to -25*	60	35.10 ± 10.31	25.55 ± 10.45	0.45 ± 6.91
*-24 to +24*	114	34.79 ± 10.05	35.99 ± 10.24	1.20 ± 6.38
*25 to 49*	33	38.28 ± 11.51	37.90 ± 10.4	-0.38 ± 5.94
*50 to 99*	40	36.03 ± 10.94	35.80 ± 10.91	-0.23 ± 7.29
>= 100	28	34.09 ± 11.65	36.81 ± 10.64	2.72 ± 9.72
		**RAND12 MCS**		
*<= -200*	7	47.28 ± 5.87	43.74 ± 9.05	-3.53 ± 6.21
*-199 to -100*	16	46.65 ± 13.15	38.56 ± 11.92	-8.09 ± 14.05
*-99 to -25*	60	47.66 ± 11.96	46.92 ± 11.38	-0.74 ± 7.87
*-24 to +24*	114	49.47 ± 11.14	49.67 ± 10.91	0.19 ± 8.55
*25 to 49*	33	47.74 ± 11.38	48.82 ± 10.86	1.08 ± 7.33
*50 to 99*	40	46.85 ± 11.22	48.38 ± 10.52	1.54 ± 8.71
>= 100	28	50.27 ± 12.16	51.37 ± 11.06	1.40 ± 9.43

### HRQL responsiveness to change

Overall, the magnitude of change in the HRQL scores were larger for subjects who improved compared to subjects who deteriorated over the 6-week period, with the exception of the 6 MW data, which showed the opposite trend (Table 5). Importantly, however, fewer subjects were classified as having deteriorated over the follow-up period, irrespective of the external criterion used (Tables 3, 4, 5). As expected, relatively small changes in HRQL scores occurred in subjects who were classified as having not changed during the follow-up period on the external clinical criterions.

Similar to the raw HRQL change scores, the magnitude of the responsiveness indices were influenced by the direction of clinical change (Tables 6, 7, 8). Overall, the responsiveness indices were larger for subjects who improved during the follow-up period compared to individuals who deteriorated, irrespective of the responsiveness index calculated. For example, the T-statistic for the EQ-5D–US Scoring system for subjects who improved substantially (i.e., +5, +6, +7) on the global rating of change criterion was 2.13 compared to only 1.00 for subjects who substantially deteriorated (i.e., -5,-6,-7) (Table 7). Similar trends were observed in the other HRQL measures.

**Table 6:** Responsiveness statistics and relative ranking of selected HRQL measures according to change on the external criterion: New York Heart Association

**Improved +2 NYHA Classes**	**n**	**T-Statistic (Rank)**	**Effect Size (Rank)**	**Guyatt's Responsiveness Statistic (Rank)**	**Standardized Response Mean (Rank)**	**Median Rank**
EQ5D–US Scoring	2	1.00 (3)	0.36 (3)	0.31 (4)	0.80 (3)	3.00
EQ5D–UK Scoring	2	1.00 (3)	0.21 (6)	0.20 (6)	0.80 (3)	4.50
EQ–VAS	2	4.33 (1)	0.51 (2)	0.40 (3)	3.07 (1)	1.50
KCCQ Overall Summary Score	2	0.64 (5)	3.84 (1)	0.56 (2)	0.45 (5)	3.50
KCCQ Clinical Summary Score	2	0.22(6)	0.34 (4)	0.25 (5)	0.15 (6)	5.50
RAND12 PCS	2	-1.17 (7)	-0.28 (7)	-0.43 (7)	-0.83 (7)	7.00
RAND12 MCS	2	1.54 (2)	0.25 (5)	0.67 (1)	1.09 (2)	2.00
**Improved +1 NYHA Classes**						
EQ5D–US Scoring	50	0.80 (4)	0.06 (5)	0.08 (5)	0.08 (5)	5.00
EQ5D–UK Scoring	50	0.73 (5)	0.08 (4)	0.10 (4)	0.11 (4)	4.00
EQ–VAS	46	0.31 (6)	0.05 (6)	0.07 (6)	0.05 (6)	6.00
KCCQ Overall Summary Score	50	2.16 (2)	0.20 (2)	0.42 (2)	0.31 (2)	2.00
KCCQ Clinical Summary Score	50	2.30 (1)	0.22 (1)	0.44 (1)	0.32 (1)	1.00
RAND12 PCS	50	1.07 (3)	0.13 (3)	0.22 (3)	0.15 (3)	3.00
RAND12 MCS		-0.67 (7)	-0.10 (7)	-0.12 (7)	-0.10 (7)	7.00
**No Change in NHYA Class**						
EQ5D–US Scoring	206	3.43	0.17	0.23	0.23	-
EQ5D–UK Scoring	206	3.45	0.19	0.25	0.25	-
EQ–VAS	198	1.27	0.07	0.09	0.09	-
KCCQ Overall Summary Score	206	2.46	0.08	0.17	0.17	-
KCCQ Clinical Summary Score	206	2.05	0.07	0.14	0.14	-
RAND12 PCS	206	1.97	0.08	0.14	0.14	-
RAND12 MCS	206	0.71	0.04	0.05	0.05	-
**Deteriorated -1 NYHA Classes**						
EQ5D–US Scoring	40	0.26 (5)	0.06 (6)	0.08 (6)	0.08 (6)	6.00
EQ5D–UK Scoring	40	0.12 (4)	0.00 (4)	0.00 (4)	0.00 (4)	4.00
EQ–VAS	37	0.88 (7)	0.12 (7)	0.17 (7)	0.14 (7)	7.00
KCCQ Overall Summary Score	40	-2.12 (2)	-0.18 (2)	-0.38 (2)	-0.33 (2)	2.00
KCCQ Clinical Summary Score	40	-2.80 (1)	-0.23 (1)	-0.44 (1)	-0.44 (1)	1.00
RAND12 PCS	40	0.31 (6)	0.03 (5)	0.05 (5)	0.05 (5)	5.00
RAND12 MCS	40	-1.41 (3)	-0.18 (3)	-0.26 (3)	-0.22 (3)	3.00
**Deteriorated -2 NYHA Classes**	0	Not Calculated Due to No subjects				

**Table 7:** Responsiveness statistics and relative ranking of selected HRQL measures according to change on the external criterion: Global Rating of Change

**Global Change Score – Substantial Increase +5,+6,+7**	**n**	**T-Statistic (Rank)**	**Effect Size (Rank)**	**Guyatt's Responsiveness Statistic (Rank)**	**Standardized Response Mean (Rank)**	**Median Rank**
EQ5D–US Scoring	7	2.13 (4)	0.78 (2)	1.08 (6)	0.82 (5)	4.50
EQ5D–UK Scoring	7	2.10 (5)	0.75 (3)	1.11 (5)	0.81 (6)	5.00
EQ–VAS	5	1.93 (6)	0.56 (6)	1.21 (4)	0.86 (4)	5.00
KCCQ Overall Summary Score	7	3.03 (1)	0.65 (4)	1.82 (1)	1.14 (1)	1.00
KCCQ Clinical Summary Score	7	2.86 (3)	0.57 (5)	1.65 (3)	1.08 (3)	3.00
RAND12 PCS	7	2.98 (2)	0.93 (1)	1.82 (2)	1.12 (2)	2.00
RAND12 MCS	7	-1.31 (7)	-0.40 (7)	-0.64 (7)	-0.50 (7)	7.00
**Global Change Score – Moderate Increase +2,+3,+4**						
EQ5D–US Scoring	53	1.37 (4)	0.13 (5)	0.15 (7)	0.17 (4)	4.50
EQ5D–UK Scoring	53	1.23 (5)	0.13 (6)	0.16 (5)	0.15 (5)	5.00
EQ–VAS	51	0.89 (7)	0.14 (4)	0.18 (4)	0.12 (7)	5.50
KCCQ Overall Summary Score	53	2.15 (2)	0.23 (2)	0.43 (1)	0.30 (2)	2.00
KCCQ Clinical Summary Score	53	1.45 (3)	0.16 (3)	0.30 (3)	0.20 (3)	3.00
RAND12 PCS	53	0.93 (6)	0.09 (7)	0.16 (6)	0.13 (6)	6.00
RAND12 MCS	53	2.20 (1)	0.28 (1)	0.39 (2)	0.30 (1)	1.00
**Global Change Score – No Change -1,0,+1**						
EQ5D–US Scoring	206	2.94	0.17	0.23	0.23	-
EQ5D–UK Scoring	206	3.00	0.15	0.21	0.21	-
EQ–VAS	196	1.49	0.08	0.11	0.11	-
KCCQ Overall Summary Score	206	2.02	0.06	0.14	0.14	-
KCCQ Clinical Summary Score	206	1.80	0.06	0.13	0.13	-
RAND12 PCS	206	1.85	0.08	0.13	0.13	-
RAND12 MCS	206	-0.83	-0.04	-0.06	-0.06	-
**Global Change Score – Moderate Deterioration -2,-3,-4**						
EQ5D–US Scoring	30	-0.31 (6)	-0.07 (6)	-0.08 (6)	-0.09 (6)	6.00
EQ5D–UK Scoring	30	-0.29 (7)	-0.05 (7)	-0.05 (7)	-0.05 (7)	7.00
EQ–VAS	29	-0.91 (5)	-0.14 (5)	-0.17 (5)	-0.17 (5)	5.00
KCCQ Overall Summary Score	30	-3.17 (1)	-0.32 (1)	-0.62 (1)	-0.58 (1)	1.00
KCCQ Clinical Summary Score	30	-2.62 (2)	-0.28 (2)	-0.51 (2)	-0.48 (2)	2.00
RAND12 PCS	30	-1.59 (3)	-0.21 (4)	-0.26 (4)	-0.29(3)	3.50
RAND12 MCS	30	-1.40 (4)	-0.23 (3)	-0.30 (3)	-0.25 (4)	3.50
**Global Change Score – Substantial Deterioration -5,-6,-7**						
EQ5D–US Scoring	2	-1.00 (4)	-0.16 (5)	-0.23 (6)	-0.60 (6)	5.50
EQ5D–UK Scoring	2	-1.00 (4)	-0.15 (6)	-0.26 (5)	-0.71 (4)	4.50
EQ–VAS	2	-1.67 (2)	-3.54 (1)	-1.57 (1)	-1.18 (2)	1.50
KCCQ Overall Summary Score	2	-4.71 (1)	-0.97 (2)	-0.79 (2)	-3.33 (1)	1.50
KCCQ Clinical Summary Score	2	-1.60 (3)	-0.57 (3)	-0.77 (3)	-1.13 (3)	3.00
RAND12 PCS	2	0.47 (7)	1.07 (7)	0.23 (7)	0.33 (7)	7.00
RAND12 MCS	2	-1.00 (4)	-0.21 (4)	-0.26 (4)	-0.71 (5)	4.00

**Table 8:** Responsiveness statistics and relative ranking of selected HRQL measures according to change on the external criterion: Six-Minute Walk Test

**6 Minute Walk – Deteriorated <= -200**	**n**	**T-Statistic (Rank)**	**Effect Size (Rank)**	**Guyatt's Responsiveness Statistic (Rank)**	**Standardized Response Mean (Rank)**	**Median Rank**
EQ5D–US Scoring	7	-0.45 (5)	-0.18 (5)	-0.18 (5)	-0.22 (5)	5.00
EQ5D–UK Scoring	7	-0.06 (6)	0.00 (6)	0.00 (6)	0.00 (6)	6.00
EQ–VAS	7	-1.23 (4)	-0.65 (1)	-0.55 (3)	-0.46 (4)	3.50
KCCQ Overall Summary Score	7	-1.56 (2)	-0.34 (3)	-0.71 (2)	-0.59 (2)	2.00
KCCQ Clinical Summary Score	7	-1.88 (1)	-0.32 (4)	-0.72 (1)	-0.71 (1)	1.00
RAND12 PCS Score	7	0.08 (7)	0.03 (7)	0.04 (7)	0.03 (7)	7.00
RAND12 MCS Score	7	-1.51 (3)	-0.60 (2)	-0.41 (4)	-0.57 (3)	3.00
**6 Minute Walk – Deteriorated -199 to -100**						
EQ5D–US Scoring	16	0.59 (5)	0.08 (5)	0.18 (6)	0.17 (6)	5.50
EQ5D–UK Scoring	16	0.59 (5)	0.09 (6)	0.18 (5)	0.15 (5)	5.00
EQ–VAS	15	-1.44 (3)	-0.31 (2)	-0.49 (2)	-0.37 (3)	2.50
KCCQ Overall Summary Score	16	-3.23 (1)	-0.20 (3)	-0.46 (3)	-0.81 (1)	2.00
KCCQ Clinical Summary Score	16	-1.23 (4)	-0.13 (4)	-0.26 (4)	-0.31 (4)	4.00
RAND12 PCS	16	1.74 (7)	0.20 (7)	0.38 (7)	0.43 (7)	7.00
RAND12 MCS	16	-2.30 (2)	-0.62 (1)	-0.95 (1)	-0.58 (2)	1.50
**6 Minute Walk – Deteriorated -99 to -25**						
EQ5D–US Scoring	60	1.83 (6)	0.24 (7)	0.36 (7)	0.27 (7)	7.00
EQ5D–UK Scoring	60	1.83 (6)	0.23 (6)	0.35 (6)	0.26 (6)	6.00
EQ–VAS	58	0.92 (5)	0.12 (5)	0.13 (5)	0.12 (5)	5.00
KCCQ Overall Summary Score	60	0.04 (2)	0.00 (2)	0.01 (2)	0.01 (2)	2.00
KCCQ Clinical Summary Score	60	0.31 (3)	0.02 (3)	0.05 (3)	0.04 (3)	3.00
RAND12 PCS	60	0.51 (4)	0.04 (4)	0.07 (4)	0.07 (4)	4.00
RAND12 MCS	60	-0.73 (1)	-0.06 (1)	-0.09 (1)	-0.09 (1)	1.00
**6 Minute Walk – No Change -24 to 24**						
EQ5D–US Scoring	114	0.58	0.00	0.00	0.00	-
EQ5D–UK Scoring	114	0.58	0.04	0.06	0.06	-
EQ–VAS	109	0.74	0.07	0.07	0.07	-
KCCQ Overall Summary Score	114	1.44	0.06	0.13	0.13	-
KCCQ Clinical Summary Score	114	0.59	0.03	0.06	0.06	-
RAND12 PCS	114	2.01	0.12	0.19	0.19	-
RAND12 MCS	114	0.24	0.02	0.02	0.02	-
**6 Minute Walk – Improved +25 to +49**						
EQ5D–US Scoring	33	1.66 (1)	0.25 (1)	0.36 (1)	0.31 (1)	1.00
EQ5D–UK Scoring	33	1.66 (1)	0.22 (2)	0.29 (2)	0.29 (2)	2.00
EQ–VAS	31	1.06 (3)	0.14 (3)	0.14 (3)	0.19 (3)	3.00
KCCQ Overall Summary Score	33	0.08 (5)	0.01 (5)	0.02 (5)	0.01 (5)	5.00
KCCQ Clinical Summary Score	33	-0.01 (6)	0.00 (6)	0.00 (6)	0.00 (6)	6.00
RAND12 PCS	33	-0.04 (7)	-0.03 (7)	-0.06 (7)	-0.06 (7)	7.00
RAND12 MCS	33	0.85 (4)	0.09 (4)	0.13 (4)	0.15 (4)	4.00
**6 Minute Walk – Improved +50 to +99**						
EQ5D–US Scoring	40	2.10 (1)	0.28 (2)	0.45 (2)	0.36 (1)	1.50
EQ5D–UK Scoring	40	2.10 (1)	0.29 (1)	0.47 (1)	0.35 (2)	1.00
EQ–VAS	38	1.35 (5)	0.16 (4)	0.22 (5)	0.22 (5)	5.00
KCCQ Overall Summary Score	40	1.73 (4)	0.14 (5)	0.34 (4)	0.22 (4)	4.00
KCCQ Clinical Summary Score	40	1.92 (3)	0.16 (3)	0.36 (3)	0.30 (3)	3.00
RAND12 PCS	40	-0.20 (7)	-0.02 (7)	-0.04 (7)	-0.03 (7)	7.00
RAND12 MCS	40	1.12 (6)	0.14 (6)	0.18 (6)	0.18 (6)	6.00
**6 Minute Walk – Improved >= +100**						
EQ5D–US Scoring	28	1.68 (4)	0.25 (4)	0.36 (4)	0.36 (4)	4.00
EQ5D–UK Scoring	28	1.68 (4)	0.21 (6)	0.29 (6)	0.31 (5)	5.50
EQ–VAS	27	1.93 (3)	0.26 (3)	0.33 (5)	0.37 (3)	3.00
KCCQ Overall Summary Score	28	3.87 (1)	0.48 (1)	0.94 (1)	0.73 (1)	1.00
KCCQ Clinical Summary Score	28	3.09 (2)	0.37 (2)	0.71 (2)	0.58 (2)	2.00
RAND12 PCS	28	1.48 (6)	0.23 (5)	0.43 (3)	0.28 (6)	5.50
RAND12 MCS	28	0.79 (7)	0.12 (7)	0.16 (7)	0.15 (7)	7.00

**Figure 1:**
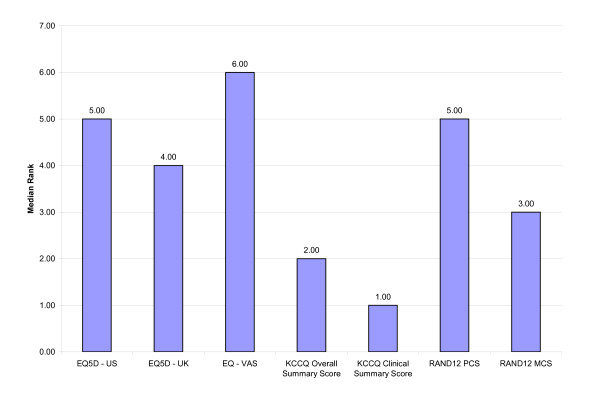
Overall Relative Rank for Selected HRQL Measures According to External Clinical Criterion: New York Heart Association.

**Figure 2:**
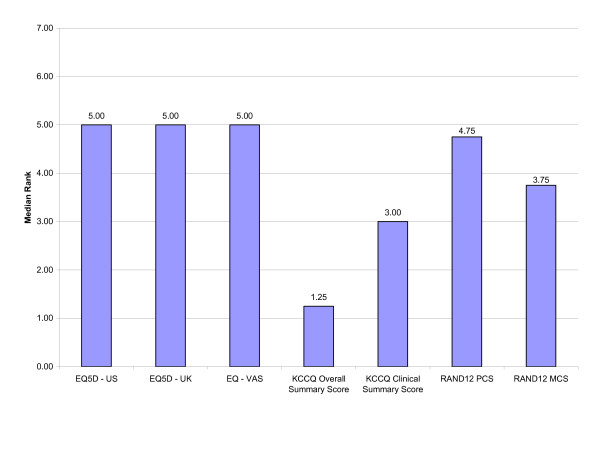
Overall Relative Rank for Selected HRQL Measures According to External Clinical Criterion: Global Rating of Change.

**Figure 3:**
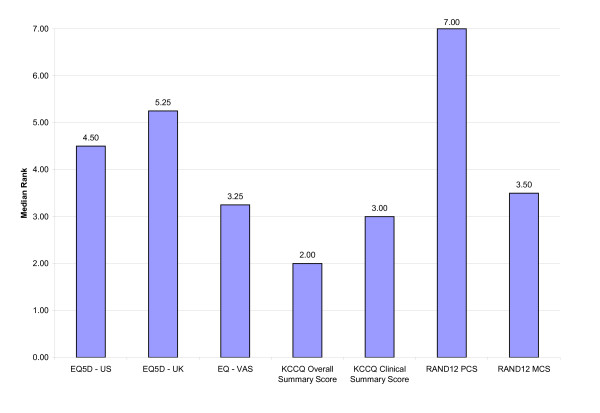
Overall Relative Rank for Selected HRQL Measures According to External Clinical Criterion: Six-Minute Walk Test.

In general, the relative ranking of the HRQL measure within each responsiveness index was similar, regardless of the responsiveness indices used (Tables 6, 7, 8). Irrespective of the responsiveness index used, the KCCQ Clinical Summary Score and Overall Summary Score were consistently ranked as the most responsive measures (Figures 1, 2, 3). Interestingly, the KCCQ Overall Summary Score was ranked as the most responsive measure according to the global rating of change (Figure 2) and 6 MW (Figure 3), but not with respect to the NYHA classification (Figure 1). This is not surprising as the NYHA Classification is focused mainly on the 'clinical' aspects of heart failure (symptoms and function). As a result, this criterion is more attuned to the KCCQ Clinical Summary Score compared to the KCCQ Overall Summary Score, which also includes the domains of social function and quality of life.

Although small differences existed, the relative ranking of the generic HRQL measures across the responsiveness indices were similar and generally differed by only one rank position. For example, the EQ-5D–US Scoring system for subjects who improved +2 NYHA classes had a relative ranking of '3' using the T-statistic, ES and GRS and a rank of '4' using the SRM. Thus, the relative responsiveness of a disease-specific versus generic HRQL measure was not substantively influenced by the choice of responsiveness index. Differences did exist, however, in the relative ranking of generic HRQL measures according to the external clinical criterion of change used (Tables 6, 7, 8; Figures 1, 2, 3). The observed differences were relatively small, however. In general, the EQ-5D scoring systems and the RAND12 PCS scores were not as responsive as the RAND12 MCS score or the KCCQ scores.

## Discussion

In this study, we found that the disease specific KCCQ measure was more responsive to underlying clinical change than either the generic EQ-5D or RAND12 measures. The greater responsiveness of the KCCQ was consistent across all four responsiveness indices used and across the three external clinical criteria of change. Importantly, we also found that the methods and definitions used to define *true *underlying change can have a major influence on the perceived responsiveness of a HRQL measure [19,20], particularly for the generic HRQL measures. Our results showed that the generic HRQL measures may be highly responsive when compared to one clinical anchor yet less responsive with a different clinical anchor. These effects were less apparent with the KCCQ. As a result, different methods used to judge whether an important change has occurred in a patient can lead to different conclusions regarding the responsiveness of a HRQL measure. These are important results, given the broad range of approaches that may be applied in the assessment of responsiveness [9].

It is important to understand and interpret how changes in the HRQL scores over time reflect *true *underlying change in patients with heart failure. Clinicians are interested in determining if the difference in HRQL scores over time signifies a trivial, small but clinically important, moderate, or a large change in HRQL [5,21]. This information is critical in guiding clinical decisions with respect to the patients' management. Furthermore, in the clinical trial setting, this information is equally important to determine if there is sufficient evidence to support a new treatment modality.

The high responsiveness of the KCCQ may have been expected as specific measures, by design, typically have very strong content validity for a specific disease or population. It is generally accepted that when true change occurs in the setting of a clinical trial, disease-specific measures are more responsive to this change as compared to generic measures of HRQL [22]. They are often perceived to be more clinically relevant and 'sensible' to both patients and clinicians [23]. Disease specific measures generally also explore a single domain in greater depth compared to a corresponding domain in generic measures [24].

The KCCQ, for example, specifically focuses on the impact of dyspnea, a prominent complaint for people with heart failure. In the RAND12, dyspnea could be captured, but only in much broader terms under the domain of physical functioning. Thus, disease-specific measures may be more sensitive and responsive to within-patient change as compared to generic measures [22]. This change in HRQL is often easier to identify using disease specific measures since changes observed on the measure are often more closely associated with changes in clinical measurements which are familiar to clinicians [25]. In addition, the lower responsiveness of the generic measures may also be due, in part, to the presence of other competing comorbidities that can influence generic measures, other than the severity or changes in the patient's heart failure. As a result, the stronger responsiveness of the KCCQ may have been expected.

While our data would suggest that the use of a disease-specific measure, like the KCCQ, may provide the best opportunity to capture small but highly relevant clinical changes in heart failure patients, not all changes in HRQL may be captured with the use of a disease-specific measure, as often the overall effects of a new treatment may not be fully known. For example, we were intrigued by the relative performance of the physical and mental health summary scores of the RAND12. Although heart failure is often perceived largely as a physical disease, the RAND12 PCS Score performed very poorly compared to the other HRQL measures. The reason for the lower responsiveness of the RAND12 PCS is not known but may be related to the underlying health status of the study population. RAND12 PCS scores were, on average, 1.5 standard deviations below the standardized US population mean at baseline indicating that the subjects had significant physical deficits (Table 2). As a result, it is possible that subjects were too limited, physically, to change over the 6-week follow-up period significantly. Alternatively, since patients recruited in the study were from the outpatient setting, patients would be expected to have 'relatively' stable heart failure symptoms. As a result, significant physical changes may not necessarily be expected over the 6-week period. Interestingly, however, the RAND12 MCS was relatively more responsive to changes in heart failure status. The impact of mental health and its monitoring in patients with heart failure warrants further investigation [26].

Also of note was that the relative responsiveness of the HRQL measures depended on the direction of clinical change. Overall, the HRQL measures were more responsive to improved clinical status as compared to deteriorating clinical status. This may be related to the fact that heart failure patients in this study were quite severely affected by their disease and had substantial deficits in their HRQL. As a result, HRQL measures may be susceptible to 'floor effects' in this population, which may limit their ability to capture deterioration in clinical status. The observation that no patient deteriorated by 2 NYHA classes further supports this hypothesis. Thus, not only can the responsiveness indices and external criterion standards influence instrument responsiveness, but also it is important to consider the population studied.

Irrespective of the scope of the HRQL measure or the direction of clinical change, the responsiveness of a particular measure may also be influenced by the responsiveness index used. Although the responsiveness indices used in this study provided similar rankings, differences did exist among the responsiveness indices when the generic HRQL measures were concerned. In this study, four commonly utilized responsiveness indices were used [9,27]. Terwee et al. have shown that there are over 30 different responsiveness calculations which have been described in the literature to identify change in a patient's HRQL [9].

Although most indices use the mean change in HRQL over time, there are significant differences in how the standard deviations or variability in the data is used in the calculation. For example, the GRS was calculated using the standard deviation of the change scores among subjects who are clinically stable, whereas the SRM uses the standard deviation of the change scores. It is therefore possible that significant differences could exist in the variability in the selected subgroups, resulting in differences in the perceived responsiveness of the HRQL measure depending upon the responsiveness index chosen.

Our results and interpretation should be considered in light of several potential limitations. First, as discussed, the ability to identify patients who truly changed during the follow-up period was subjective, as no 'gold standard' exists for patients with heart failure. We did apply, however, the NYHA classification system, physician global rating of change assessment, and 6 MW tests, which are well validated and common methods to identify change in clinical status in patients with heart failure [2,17,18]. Furthermore, the same cardiologist evaluated subjects at both the baseline and the 6-week follow-up improving internal consistency of these change ratings and may provide the most appropriate method for validating the HRQL measures ability to identify *true *clinical change in this population [2]. Second, the categorical cut points used in the 6 MW data to indicate the magnitude of clinical change may have affected the responsiveness results. We did reanalyze the data using different categorical cut points, but obtained the same relative ranking of the HRQL measures. As a result, it is unlikely that changes in the cut points for the clinical change categories for the 6 MW data would significantly alter our results. Third, fifteen subjects were missing either baseline or 6-week EQ-VAS scores. It is possible that restricting the sample to subjects who had complete responses on all the HRQL measures may have changed the relative ranking of the HRQL measures. We feel this is unlikely, however, as the majority of subjects missing EQ-VAS scores were classified as not changing over the 6-weeks according to the external criterions. Thus, the effect of the missing data on the responsiveness rankings would be minimal. Fourth, we chose to evaluate four of the more commonly used responsiveness indices in this study. Numerous other responsiveness indices have been reported in the literature. While it may be possible that different results would have been observed if other responsiveness indices were used, we think that is unlikely. Finally, the duration of follow-up was relatively short and may have affected the estimated responsiveness of the HRQL measures. Each HRQL measure uses a different period ranging from 'today' (EQ-5D) to 'within the past 4 weeks' (RAND12) to assess the patients' health status. The 6-week follow-up period was initially chosen to improve the recall accuracy of the cardiologist evaluating the patient, yet long enough to allow meaningful clinical change to occur [2].

With these limitations in mind, we believe this study highlights the importance of considering the measurement properties and instrument content in selecting HRQL measures for clinical trials. It is important to have a measure capable of detecting small but highly relevant and important changes in HRQL, especially when the HRQL outcome of interest represents the primary outcome or main secondary outcome of the trial. Furthermore, in the design of a clinical trial, researchers must be confident that the HRQL measure is responsive to the minimal importance difference that was hypothesized during the study design. The sample size required and power of the study will be directly related to the minimal importance difference that the researchers wish to detect. However, while disease specific measure may be considered as the first choice for a primary HRQL outcome, a combination with a generic HRQL measure may still be desirable to fully assess HRQL outcomes in clinical trial settings. Further, if the therapy under consideration will be evaluated under a cost-effectiveness framework, it would be desirable to include a measure compatible with that framework, such as a utility-based measure [28].

## Conclusion

We found the disease specific measure, the KCCQ, was the most responsive HRQL measure assessing change over a 6-week period, although generic measures provide information for which the KCCQ is not suitable. We noted that the responsiveness of generic HRQL measure may be affected by the responsiveness index used, as well as the selection of the external criterion to identify patients who have clinically change or remained stable.

## Competing interests

Dr. Spertus owns the copyright to the KCCQ. While he assisted in the review of the manuscript and was responsible for the original study, he did not design these analyses nor dictate the interpretation of the results. Otherwise, the authors have no conflicts of interest, including specific financial interests, non-financial interests, relationships, and affiliations relevant to the subject matter or materials discussed in the manuscript.

## Authors' contributions

DTE and JAJ participated in the conception and design of the study, analysis, and interpretation of the data, drafting of the manuscript, and revising it critically for important intellectual content. KJL participated in the analysis and interpretation of the data and revising the manuscript for critically important intellectual content. JAS participated in the conception and design of the study, acquisition of data, interpretation of the data, and revising the manuscript for critically important intellectual content. All authors read and approved the final manuscript.
